# Using Serological Proteome Analysis to Identify and Evaluate Anti-GRP78 Autoantibody as Biomarker in the Detection of Gastric Cancer

**DOI:** 10.1155/2020/9430737

**Published:** 2020-12-18

**Authors:** Jiejie Qin, Qian Yang, Hua Ye, Keyan Wang, Meng Zhang, Jicun Zhu, Xiao Wang, Liping Dai, Peng Wang, Jianying Zhang

**Affiliations:** ^1^Department of Epidemiology and Statistics, College of Public Health, Zhengzhou University, Zhengzhou 450001, Henan Province, China; ^2^State Key Laboratory of Esophageal Cancer Prevention and Treatment & Henan Key Laboratory of Tumor Epidemiology, Zhengzhou University, Zhengzhou 450001, Henan Province, China; ^3^Henan Institute of Medical and Pharmaceutical Sciences, Zhengzhou University, Zhengzhou 450052, Henan Province, China

## Abstract

The serological biomarkers as noninvasive tests are the most promising way for diagnosing gastric cancer (GC). Serological proteome analysis (SERPA) has been used to identify tumor-associated antigens (TAAs) and the corresponding autoantibodies in many studies. To explore the relationship between gastric cancer development and serum autoantibody anti-GRP78 response found by the method of SERPA with the GC cell line AGS, we included two cohorts (133 GC and 133 normal individuals in test group; 300 GC and 300 normal individuals in validation group) of patients with newly diagnosed GC for verification. All GC and normal controls were matched by age and gender. The autoantibody levels of the sera in two cohorts were measured by immunoassay. Finally, the results showed that 78-kDa glucose-regulated protein (GRP78) was identified in GC by SERPA and the level of anti-GRP78 antibody in GC was higher than that in normal individuals in the two cohorts. Receiver operating characteristic (ROC) curve analysis showed similar diagnostic value of anti-GRP78 antibody in test group (AUC: 0.718) and validation group (AUC: 0.666) to identify GC patients from normal individuals. The AUCs of anti-GRP78 autoantibody in the diagnosis of GC patients with different clinical characteristic ranged from 0.676 to 0.773 in test group and ranged from 0.645 to 0.707 in validation group. In conclusion, autoantibody against GRP78 might be a potential diagnostic biomarker. Further large-scale studies will be needed to validate and improve its performance of the sensitivity, specificity, and AUC value in distinguishing GC from other diseases.

## 1. Introduction

Gastric cancer remains a common cancer worldwide. There were more than 1,000,000 new cases in 2018 and estimated 783,000 deaths, making it the fifth most frequently diagnosed cancer and the third leading cause of cancer death [1]. The incidence rates of GC in Eastern Asia were higher than other countries [2]. In addition, because most of GC patients were diagnosed at an advanced stage and have a poor prognosis, 5-year survival rates of GC patients were very low [3]. In present, the “golden standard” for diagnosing GC is tissue biopsy, as well as endoscopy, which are not used for screening high risk individuals. Novel, reliable, and noninvasive tests were needed to improve detection of early-stage gastric cancer [4].

The immune systems of patients have the ability of recognizing antigenic changes to produce autoantibody. Specific serological autoantibodies have been observed not only in the systematic autoimmune diseases such as systemic lupus erythematosus (SLE) [5, 6] and rheumatoid arthritis (RA) [7], but also in cancers [8, 9]. The mechanism of producing autoantibodies is still poorly understood. However, autoantibodies against tumor-associated antigens (TAAs) are detectable in cancer sera because of their stability in serum and have been used as diagnostic and prognostic biomarkers for cancers in many studies [10–13]. At present, some studies have reported potential biomarkers for the diagnosis of gastric cancer, such as 14-3-3zeta [14], and GRP78 [15]. It is important for identification of novel antibodies to improve the performance in detection of gastric cancer.

Serological proteome analysis (SERPA) is one of proteome approaches and has been widely used to screen biomarkers in various diseases [16–18]. The whole protein lysates were obtained from the tumor cells or tissues from cancer patients, separated by two-dimensional gel electrophoresis, transferred onto NC membrane, and immunodetected by cancer sera and normal sera to identify the candidate TAAs by mass spectrometry (MS) [19]. Tsunemi et al. identified GRP78 as a potential biomarker in Japanese with gastric cancer by SERPA [15] and Liu et al. reported 14-3-3zeta as a potential antigen in the detection of gastric cancer [20]. In this study, we used SERPA to identify the candidate TAAs and the corresponding autoantibody in the detection of Chinese with GC in the current study; then the diagnostic values of the candidate TAAs anti-GRP78 were further estimated in Chinese with GC.

## 2. Materials and Methods

### 2.1. Participants

The study included two phases to identify and validate new autoantibodies in sera, including discovery phase and validation phase. The detailed flowchart of the study is shown in Supplementary Figure 1. In the discovery phase, 104 gastric cancer and 54 normal health sera were selected from the lab biobank in the Henan Key Laboratory of Tumor Epidemiology to explore whether interesting autoantibodies were in GC but not in normal sera in WB. The 104 GC sera were collected in the First Hospital affiliated to Zhengzhou University in 2010 and 54 normal individuals were matched with GC patients by age and gender. In addition, SERPA and MS detection was used to identify the candidate TAAs and the corresponding autoantibody.

In the validation phase, a total of 866 participants were included in the validation phase in the current study and were randomly divided into two group, including test group and validation group. 133 GC patients and 133 normal individuals were used as test group, and 300 GC patients and 300 normal individuals were used as validation group. All 433 GC sera were collected in the First Hospital affiliated to Zhengzhou University from January 2016 to June 2017 before these patients received surgery treatment, radiotherapy, chemotherapy, and other treatments. All the normal sera were matched with the cases by age and gender and selected from the biobank. This study was approved by the institutional review boards of Zhengzhou University, and all participants were informed of the content.

### 2.2. Cell Line Culture

The human stomach adenocarcinoma cell line (AGS) cells were purchased from the American Type Culture Collection (ATCC, Manassas, VA, USA) and cultured in F-12K medium (Gibco, Carlsbad, CA) with 10% fetal bovine serum (FBS). AGS cells grown in 75 cm^2^ Falcon tissue culture flasks. When cells covered the 95% flask, F-12K medium was used to rinse the cells once. Then, trypsin (Gibco, Carlsbad, CA) was incubated with cells to remove cells from the flask. Finally, cells were collected in a centrifuge tube for further study.

### 2.3. Western Blotting

For screening the antibody-positive sera, the whole AGS cells were lysed in Laemmli's sample buffer, and the proteins were separated in 10% SDS-polyacrylamide gels (SDS-PAGE gels) and then transferred into nitrocellulose (NC) membrane (Osmonics Inc., MA) for western blotting analysis. The membrane with proteins was cut into 0.3 cm wide strips. After blocking with 3% nonfat milk prepared in PBST, the NC membrane strips were individually incubated with human serum samples (including 104 gastric cancer and 54 normal health sera) diluted at 1 : 100 in 3% milk solution. Horseradish peroxidase (HRP)-conjugated goat anti-human IgG (Thermo Fisher Scientific, Waltham, MA) was used as secondary antibody at 1 : 10,000 dilution. Finally, the immunoreactive bands were detected with Enhanced Chemiluminescence (ECL) kit (Thermo Fisher Scientific, Waltham, MA).

### 2.4. Two-Dimensional Gel Electrophoresis Analysis

AGS cells were lysed in the rehydration buffer purchased from Bio-Rad. The cell lysates were vortexed at room temperature for 1 hour and then centrifuged at 16000 g for 20 min at 4°C. Then, the supernatant was collected and protein concentration was detected by the Bradford assay (Bio-Rad, Hercules, CA).

For the first dimensional gel electrophoresis (1-DE) analysis, 150 *µ*g of extracted protein was mixed with 125 *µ*l rehydration buffer applied on an immobilized pH gradient (IPG) strip (pH 3–10, 7 cm) (Bio-Rad, Hercules, CA). A total of 3 IPG strips were rehydrated in the current study and were run in parallel under identical conditions. Isoelectric focusing (IEF) was performed at 50 mA per gel, 250 V for 30 min, followed by 4,000 V for 2.5 hours, and additional 4,000 V for 25,000 V-hour. Three strips were then immediately stored at −80°C and subsequently used for 2-DE analysis with 10% SDS-PAGE.

For the second dimensional gel electrophoresis (2-DE) analysis, the proteins on 3 IPG strips were separated on 10% SDS-PAGE gels, respectively. One 2-DE gel was dyed in Coomassie brilliant blue dye and the proteins from the other two SDS-PAGE gels were transferred into two NC membranes, respectively. After blocking with 5% notfat milk, the two NC membranes were incubated with the mixture of five antibody-positive sera from patients with GC and the pool of five normal individual sera (each diluted at 1 : 500), respectively. HRP-conjugated goat anti-human IgG was used as secondary antibody at 1 : 10,000 dilution. The immunoreactive spots were detected with ECL kit. After comparing and identifying the protein spots in the 2-DE gel corresponding to the immunoreactive spots in the 2-DE western blotting, a 78-kDa protein spot of interest in the 2-DE gel was excised and digested to perform matrix-assisted laser desorption/ionization time-of-flight mass spectrometry (MALDI-TOF/TOF MS) analysis by the company (Applied Protein Technology, Shanghai, China).

### 2.5. Indirect Enzyme-Linked Immunosorbent Assay (ELISA)

The recombinant GRP78 protein was purchased from the Cloud-Clone Company. Anti-GRP78 autoantibodies in sera were detected by ELISA as previously described [14]. Briefly, proteins were diluted in coating buffer to a final concentration of 0.25 mg/ml and then were coated on 96-well microtiter plates (Fisher Scientific, Pittsburgh, PA) at 4°C overnight. The sera at 1 : 100 dilutions were added to the antigen-coated wells and incubated for 2 hours followed by five washes with PBST. HRP-conjugated goat anti-human IgG (Santa Cruz Biotechnology Inc., Dallas, TX) at 1 : 10,000 dilution and the substrate TMB (Sigma, Ronkonkoma, NY) were used as detecting reagents. The optical density (OD) at 450 nm was used for data analysis.

### 2.6. Statistical Analysis

SPSS software (version 21.0) and Prism software (version 7.0, GraphPad) were used to analyze data. Because the serum autoantibody against GRP78 was not normally distributed (Shapiro–Wilk's test), Mann–Whitney *U* tests were used to compare differences of antibody levels between GC group and normal group. The area under the curve (AUC) with 95% confidence internal (CI) was calculated by receiver operating characteristic (ROC) curve analysis. *χ*^2^ tests were used to compare the differences of frequency between two groups. The optimal cutoff thresholds for designating positive reaction were determined, while Youden's Index is the highest and specificity is more than 90%. The sensitivity, specificity, and likelihood ratio were determined based on the optimal cutoff values. Differences were considered statistically significant when *P* < 0.05.

## 3. Results

### 3.1. Serological Proteome Analysis (SERPA)

**Figure 1:**
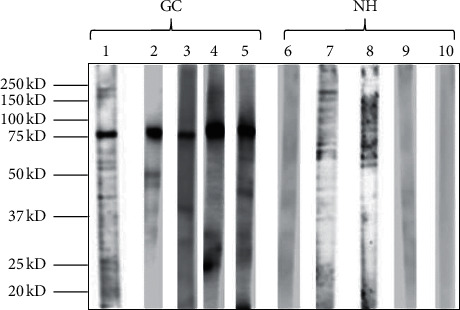
Western blotting analysis of AGS cell in GC sera and normal health sera. Lanes 1–5: GC sera contain antibodies against 78-kDa cellular proteins; lanes 6–10: normal individual sera without 78-kDa reactive band.

**Figure 2:**
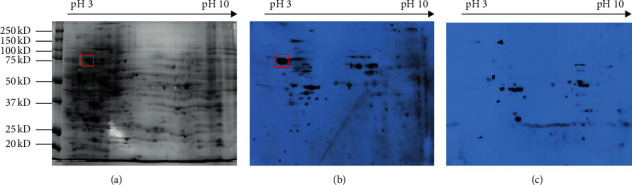
The identification of autoantibodies in sera from GC patients. (a) 2-DE protein profile of AGS cells with Coomassie brilliant blue staining. (b) 2-DE western blotting analysis of the proteins from AGS cells in the mixture of five GC sera. (c) 2-DE western blotting analysis of the proteins from AGS cells in the mixture of five normal sera.

SERPA was used to discover novel anti-TAA autoantibodies in sera from patients with GC. In the current study, the whole proteins from AGS cells were used to detect the presence of autoantibodies in 104 gastric cancer sera and 54 normal sera by western blotting analysis. The results showed that 39 out of 104 (37.5%) GC sera and 4 out of 54 (7.4%) normal sera had antibodies against an unknown protein with a molecular weight about 78-kDa (Figure 1). We next identified this GC-associated antigen by the SERPA approach. Proteins from the AGS cells were first separated by 2-DE using three gels that were run in parallel under identical condition. One of the 2-DE gels was stained with Coomassie blue (Figure 2(a)) and the other two gels were used to transfer proteins into two NC membranes. Then, the two NC membranes were incubated with a pool of five GC sera (Figure 2(b)) and a pool of five normal sera (Figure 2(c)) for western blotting analysis. Figure 2(b) showed that one immunoreactive spot migrating around 78-kDa was detected in the GC sera mixture, which was not detected in the normal sera pool (Figure 2(c)). The corresponding protein spot in the gel (Figure 1(a)) was subsequently excised and analyzed by MALDI-TOF/TOF MS. Finally, 10 potential proteins for the spot were provided in MS detection based on the protein score. However, the protein with protein score CI ≥ 95% was thought to be identified successfully. Among the 10 proteins of the spot in MS, the protein score CI of GRP-78 was 100% and its peptide count was 31. The results indicated that this protein spot matched with 78-kDa glucose-regulated protein (GRP78).

### 3.2. The Detection of Anti-GRP78 Antibody in Human Sera by ELISA

**Figure 3:**
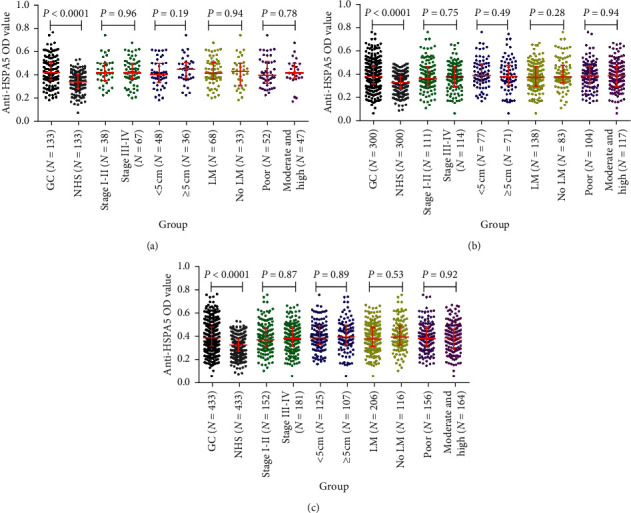
The levels of anti-GRP78 autoantibody in human sera. (a) Test cohort. (b) Validation cohort. (c) All data.

To evaluate the ability of autoantibody against GRP78 as biomarker in GC detection, the recombinant GRP78 protein was used as antigens to detect the corresponding antibody in test group (133 GC and 133 NH) and validation group (300 GC and 300 NH) by ELISA. The basic characteristics of the 433 GC and 433 NH are shown in Table 1. The gender and age distribution in GC and NH presented no difference in both test and validation group (*P* < 0.0001).  Figure 3(a) showed the antibody level of GRP78 was significantly higher in test group GC patients than that in control group. However, the levels of the anti-GRP78 antibody presented no difference in the subgroup analysis in test group (TNM : I-II vs. III-IV; tumor diameter size: <5 cm vs. ≥5 cm; lymphatic metastasis: yes vs. no; differentiation degree: poor vs. moderate and high). The same results were observed in the validation group and the combination of test and validation group (Figures 3(b) and 3(c)).

### 3.3. The Performance of Anti-GRP78 Antibody in the Diagnosis of GC

**Figure 4:**
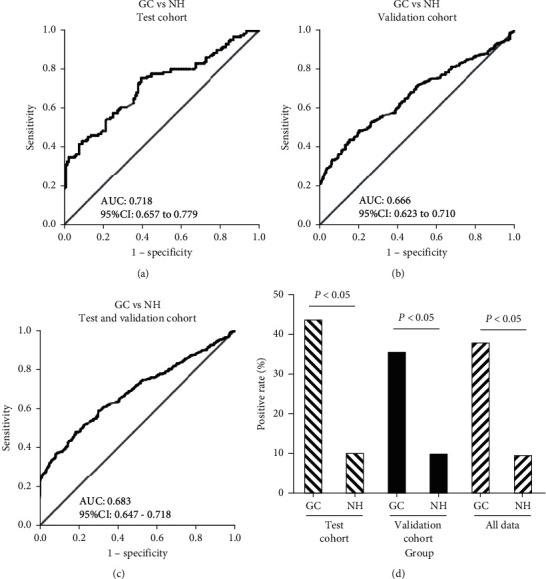
The performance of anti-GRP78 autoantibody in the diagnosis of GC. GC: gastric cancer; NH: normal human; AUC: area under the ROC curves.

The diagnostic value of anti-GRP78 antibody was evaluated by ROC curve analysis. The AUC of anti-GRP78 antibody for GC patients in test group and GC patients in validation group were 0.718 (95% CI: 0.657 to 0.779) and 0.666 (95% CI: 0.623 to 0.710) (Figures 4(a) and 4(b)). When DeLong test was used to compare the AUC of anti-GRP78 for test group and validation group, *P* value was 0.939. The result indicated that anti-GRP78 showed the similar diagnostic value in test group and validation group. When the GC patients from the two groups were integrated together, the AUC of anti-GRP78 for GC patients was 0.683 (95% CI: 0.647 to 0.718), indicating high diagnostic value for GC patients (Figure 4(c)). The sensitivity and specificity were generated according to the cutoff value with both the highest Youden's Index and more than 90% specificity from ROC curve analysis. The sensitivity and specificity of anti-GRP78 in test group were 43.6% and 9.8% (*P* < 0.05), and similar results were observed in validation group (35.3% and 9.5%, *P* < 0.05) (Figure 4(d)).

### 3.4. Performance of Anti-GRP78 Autoantibody in the Detection of GC Patients with Different Clinical Characteristics

**Figure 5:**
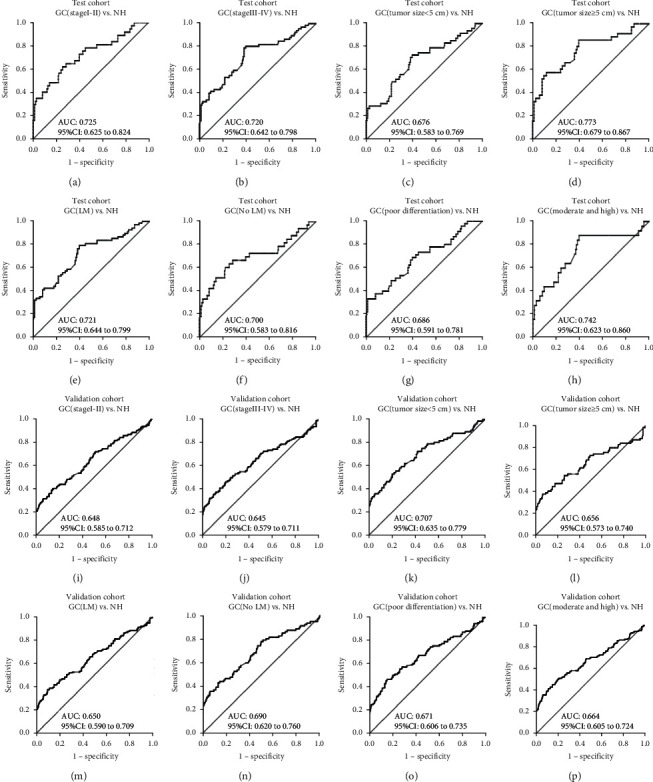
The ROC curves of anti-GRP78 antibody in the subgroup analysis. (a) Test cohort GC (stage I-II) vs. NH. (b) Test cohort GC (stage III-IV) vs. NH. (c) Test cohort GC (tumor size <5 cm) vs. NH. (d) Test cohort GC (tumor size ≥5 cm) vs. NH. (e) Test cohort GC (LM) vs. NH. (f) Test cohort GC (no LM) vs. NH. (g) Test cohort GC (poor differentiation) vs. NH. (h) Test cohort GC (moderate and high) vs. NH. (i) Validation cohort GC (stage I-II) vs. NH. (j) Validation cohort GC (stage III-IV) vs. NH. (k) Validation cohort GC (tumor size <5 cm) vs. NH. (l) Validation cohort GC (tumor size ≥5 cm) vs. NH. (m) Validation cohort GC (LM) vs. NH. (n) Validation cohort GC (no LM) vs. NH. (o) Validation cohort GC (poor differentiation) vs. NH. (p) Validation cohort GC (moderate and high) vs. NH.

All GC patients were stratified by the clinical characteristic (lymphatic metastasis, tumor size, differentiation, and TNM stage). When normal individuals were as control, ROC curves were generated for each subgroup and the results are shown in Figure 5. Anti-GRP78 was observed to significantly distinguish GC patients from normal individuals in each subgroup in both test group and validation group (Figure 5 and Table 2) (*P* < 0.05). The AUCs of subgroups in test group ranged from 0.676 to 0.773. Anti-GRP78 autoantibody presented the highest AUC of 0.773 in the subgroup of ≥5 cm GC tumor with the sensitivity of 55.6% and specificity of 90.2%. Anti-GRP78 autoantibody presented the lowest AUC of 0.676 in the subgroup of <5 cm GC tumor with the sensitivity of 29.2% and specificity of 97.0%. The similar results were observed in validation group. The AUCs of subgroups in validation group ranged from 0.645 to 0.707. The cutoff value was chosen while the Youden's Index was the largest, and the specificity was more than 90% to ensure the high specificity and AUC in GC. The frequency of anti-GRP78 autoantibody was not observed to be significantly different in comparison group in both test group and validation group (tumor size: ≥5 cm vs. <5 cm, lymphatic metastasis: yes vs. no, differentiation: poor vs. moderate and high, and stage: I + II vs. III + IV) (*P* > 0.05).

## 4. Discussion

Compared to other TAA identification approaches, such as serological analysis of recombinant cDNA expression libraries (SEREX), SERPA has some advantages. SERPA offers faster time for antigen identification and the potential for the identification of proteins with native posttranslational modifications [21]. Therefore, SERPA has been extensively used as a promising tool for identifying the repertoire of immunoreactive proteins based on 2DE gel, western blotting, and mass spectrometry. During the past decades, autoantigens were identified by SERPA in varieties of diseases, including melanoma [22], prostate cancer [19], lung cancer [16], breast cancer [23], liver fibrosis [24], type 1 diabetes [25], and primary open angle glaucoma [26]. Some studies reported that the identified GRP78 was an autoantigen in gastric cancer from a Japanese study and 14-3-3zeta was a potential biomarker in the detection gastric cancer in a study from the US by SERPA [15, 20].

In this study, in order to identify specific GC biomarker in Chinese, we have used the whole protein from AGS cells to screen sera from 105 patients with GC and 54 normal individuals by western blotting analysis. The results showed that 37.5% GC sera and 7.4% normal sera contained antibodies against an unknown protein with molecular weight of about 78-kDa. Subsequently, an immunoproteomic approach was used to identify this protein as 78-kDa glucose-regulated protein (GRP78), also known as heat shock protein family A member 5 (HSPA5) and immunoglobulin heavy chain-binding protein. GRP78 was first discovered in the endoplasmic reticulum (ER) of virtual cells and is considered as a member of the Heat Shock Protein (HSP70) family due to sharing 60% amino acid sequence homology with HSP70 [27, 28]. GRP78 is an ER chaperone and associates with other polypeptides to facilitate folding and assembly and prevent deleterious aggregations [29, 30]. In addition, under the stressful conditions, the expression levels of GRP78 were elevated extensively in cells to maintain ER stability and cell protection [29, 31].

GRP78 is related to carcinogenesis, development, and differentiation [32]. Overexpression of GRP78 was observed in tumor endothelial cell [33, 34] and in various types of cancers, such as lung [35], prostate, breast [36], melanoma [37], and hepatocellular carcinoma [38], as well as in gastric cancer [39]. However, at present, there was limited evidence for levels of autoantibody to GRP78 in sera from cancer patients. A previous study identified GRP78 protein from gastric cancer cell lines MkN-1, MkN-45, and KATOIII in Japanese patients with GC by SERPA, while it lacked the validation in a large population and reported the presence of anti-GRP78 antibody in sera from 17/60 (28.3%) patients with gastric cancer and 0/20 (0.0%) of healthy individuals on western blot analysis [15]. Furthermore, autoantibodies against GRP78 have been rarely detected as serological biomarker in the diagnosis of gastric cancer. Therefore, ELISA was used to examine the frequency of anti-GRP78 antibody in sera from GC patients and normal individuals both in test group and in validation group in the present study. The results showed that the autoantibody to GRP78 was higher in GC sera than that in normal sera. The high AUC, sensitivity, specificity, and Youden's Index were observed for anti-GRP78 antibody in the diagnosis of patients with GC, distinguishing patients from normal individuals. We also found that the expression of autoantibody to GRP78 had no association with the tumor progression, and similar results were observed in a previous study [15]. It is possible that the autoantibody takes part in tumor progression, while it is difficult for detecting the change of the autoantibody by ELISA due to its low titer in tumor progression.

All the findings indicated that anti-GRP78 might be a potential biomarker in the diagnosis of GC. However, this study just evaluated the performance of anti-GRP78 antibody to distinguish GC from normal individuals, but not other types of cancer or benign tumor. Therefore, further examination is needed to improve the utilization of anti-GRP78 autoantibody in distinguishing GC from gastritis, precancerous lesions, and some other types of cancers, including colorectal cancer, lung cancer, and prostate cancer. In addition, the methods of WB and ELISA were used to detect the reaction of antigens and the corresponding antibodies. The coimmunoprecipitation assay was necessary to certificate the direct interaction of GRP78 with its antibody in further study. Some studies reported that the levels of autoantibodies might be related to surgery therapies [19, 40]. Therefore, further study is also needed to explore if anti-GRP78 autoantibody is a prognostic biomarker and has association with clinical outcomes after therapies in a larger group of follow-up patients with GC.

## 5. Conclusions

In conclusion, autoantibody against GRP78 might be a potential diagnostic biomarker. Further large-scale studies will be needed to validate and improve its performance of the sensitivity, specificity, and AUC value in distinguishing GC from other diseases.

## Figures and Tables

**Table 1:** The basic characteristics of the participants.

Factors	Subgroup	Test group		Validation group	
		GC (*N* = 133)	NH (*N* = 133)	GC (*N* = 300)	NH (*N* = 300)
Age					
	Range	31–94	31–89	23–89	23–88
	Median	61	61	59	59
	IQR	15	16	19	18
Gender					
	Male	92 (69.2%)	92 (69.2%)	226 (75.3%)	226 (75.3%)
	Female	41 (30.8%)	41 (30.8%)	74 (24.7%)	74 (24.7%)
TNM					
	I	16 (12.0%)		55 (18.3%)	
	II	21 (15.8%)		60 (20.0%)	
	III	53 (39.8%)		85 (28.3%)	
	IV	14 (10.5%)		29 (9.7%)	
	Unknown	29 (21.8%)		71 (23.7%)	
Tumor size					
	<5 cm	48 (36.1%)		77 (25.7%)	
	≥5 cm	36 (27.1%)		71 (23.7%)	
	Unknown	49 (36.8%)		152 (50.7%)	
Lymphatic metastasis					
	Yes	68 (51.1%)		138 (46.0%)	
	No	33 (24.8%)		83 (27.7%)	
	Unknown	32 (24.1%)		79 (26.3%)	
Differentiation degree					
	Poor	52 (39.1%)		104 (34.6%)	
	Moderate	47 (35.3%)		112 (37.3%)	
	High	0 (0.0%)		5 (1.7%)	
	Unknown	34 (25.6%)		36 (25.4%)	

GC: gastric cancer; NH: normal human.

**Table 2:** The performance of anti-GRP78 antibody in the subgroup analysis.

Clinical characteristics		AUC	95% CI	Se (%)	Sp (%)	+LR	−LR	Youden's index
*Test cohort*								
TNM	Early-stage (I-II)	0.725	0.625 to 0.824	40.54	91.73	4.90	0.65	0.323
	Late-sage (III-IV)	0.720	0.642 to 0.798	40.30	91.73	4.87	0.65	0.320
Differentiation degree	Poor	0.686	0.591 to 0.781	37.78	91.73	4.57	0.68	0.295
	Moderate and high	0.742	0.623 to 0.860	44.00	90.23	4.50	0.62	0.342
LM	LM	0.721	0.644 to 0.799	42.65	90.23	4.36	0.64	0.329
	No LM	0.700	0.583 to 0.816	42.42	91.73	5.13	0.63	0.342
Tumor size	<5 cm	0.676	0.583 to 0.769	29.17	96.99	2.98	0.73	0.262
	≥5 cm	0.773	0.679 to 0.867	55.56	90.23	5.68	0.49	0.458
GC vs. NH		0.718	0.657 to 0.779	43.61	90.23	4.46	0.62	0.338
*Validation cohort*								
TNM	Early-stage (I-II)	0.648	0.585 to 0.712	33.04	90.30	3.41	0.74	0.233
	Late-sage (III-IV)	0.645	0.579 to 0.711	33.33	90.97	3.69	0.73	0.243
Differentiation degree	Poor	0.671	0.606 to 0.735	34.23	90.97	3.79	0.72	0.252
	Moderate and high	0.664	0.605 to 0.724	37.41	90.30	3.86	0.69	0.277
LM	LM	0.650	0.590 to 0.709	35.51	90.30	3.66	0.71	0.258
	No LM	0.690	0.620 to 0.760	36.14	93.31	5.40	0.68	0.295
Tumor size	<5 cm	0.707	0.635 to 0.779	37.66	90.97	4.17	0.69	0.286
	≥5 cm	0.656	0.573 to 0.740	38.03	92.98	5.41	0.67	0.310
GC vs. NH		0.666	0.623 to 0.710	35.33	90.30	3.64	0.72	0.256

LM: lymphatic metastasis; Se: sensitivity; Sp: specificity; +LR: positive likelihood ratio; −LR: negative likelihood ratio.

## Data Availability

Data are available upon request to the corresponding author.

## Supplementary Materials

Supplementary Materials Supplementary Figure 1: the flowchart of the study.
