# Supplementary figures and images for: Using Serological Proteome Analysis to Identify and Evaluate Anti-GRP78 Autoantibody as Biomarker in the Detection of Gastric Cancer
Source: J Oncol. 2020 Dec 18;2020:9430737. doi: 10.1155/2020/9430737 (PMC7762641; doi:10.1155/2020/9430737)

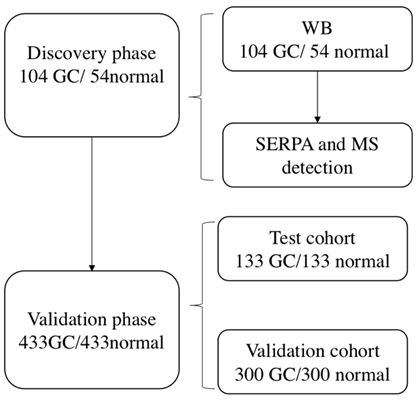


Supplementary figure 1 the flowchart of the study

Supplement: Supplementary Materials — Supplementary Figure 1: the flowchart of the study. [file 9430737.f1.docx]
